# Risk of reoperation for recurrent ptosis following intraocular surgery

**DOI:** 10.1038/s41433-025-03883-2

**Published:** 2025-06-16

**Authors:** Itay Nitzan, Tehila Shalmov, Shoham Kubovsky, Nadav Shemesh, Jaime Levy, Zvi Gur

**Affiliations:** Department of Ophthalmology, Hadassah Medical Organization and Faculty of Medicine, Hebrew University of Jerusalem, Jerusalem, Israel

**Keywords:** Epidemiology, Outcomes research

Acquired blepharoptosis (ptosis) is the abnormal drooping of the upper eyelid, potentially impairing vision and quality of life. While surgical repair is generally effective, recurrence remains a concern, influenced by patient factors, surgical technique, and ocular comorbidities [1, 2]. Intraocular surgery is a known cause of new-onset ptosis, due to factors like speculum use, bridle sutures, anaesthetic toxicity, and eyelid traction [3, 4]. Despite advances in surgical technique, the long-term effect of intraocular surgery on previously repaired ptosis has not been well studied. If intraocular surgery can induce ptosis in anatomically intact eyelids, it may similarly compromise the integrity of prior repairs, increasing the risk of recurrence and reoperation.

We conducted a retrospective cohort study using the TriNetX research network (Supplement Material) [5]. Patients aged ≥50 years who underwent ptosis repair were included and grouped into two cohorts: the Ptosis Repair with Intraocular Surgery (PRIOS) cohort, comprising patients who subsequently underwent their first intraocular surgery within 1 month to 10 years after ptosis repair, and the Ptosis Repair Only (PRO) cohort, comprising patients with no recorded intraocular surgery during the 10 years after ptosis repair. Patients with neurologic, traumatic, or congenital ptosis were excluded. The primary outcome was reoperation for recurrent ptosis.

A total of 1602 patients were included in the PRIOS cohort, and 12,093 in the PRO cohort before matching. After 1:1 propensity score matching, 1598 patients were retained in each group (Table 1). The mean follow-up time was 597.1 days (SD, 227.5) in the PRIOS cohort and 543.7 days (SD, 265.7) in the PRO cohort. At 2 years, reoperation for recurrent ptosis occurred in 38 vs. 16 patients, corresponding to incidence rates of 14.5 and 6.7 per 1000 person-years, respectively. The absolute risk of reoperation was 2.4% in PRIOS compared with 1.0% in PRO (risk difference, 1.4%; 95% CI, 0.5–2.3%; *p* = 0.003). Kaplan–Meier analysis demonstrated a significantly higher cumulative incidence of reoperation in the PRIOS cohort, with early separation of the curves (Fig. 1). The hazard ratio (HR) for reoperation was 2.16 (95% CI, 1.21–3.88). The E-value was 3.74 for the HR and 1.70 for the lower bound. The association remained significant at 5 and 10 years, with reoperation rates of 12.3 vs. 6.3 and 11.0 vs. 7.0 per 1000 person-years in the PRIOS and PRO cohorts, respectively. Hazard ratios were 1.98 (95% CI, 1.27–3.10) at 5 years and 1.67 (95% CI, 1.12–2.48) at 10 years, with corresponding E-values of 3.37 and 2.73. Kaplan–Meier curves for both timepoints are presented in the Supplemental Material.

**Fig. 1:**
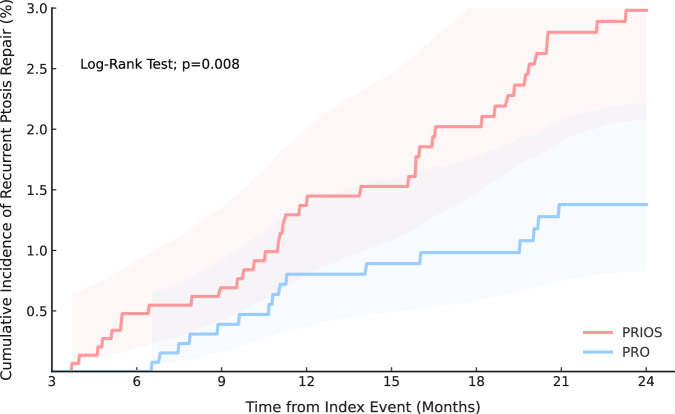
Kaplan–Meier analysis of cumulative incidence of reoperation for recurrent ptosis. This Kaplan–Meier curve depicts the cumulative incidence of reoperation for recurrent ptosis in the PRIOS (Ptosis Repair with Subsequent Intraocular Surgery) and PRO (Ptosis Repair Only) cohorts over time (months). The PRIOS cohort (red) demonstrated a higher cumulative incidence compared to the PRO cohort (blue), with a hazard ratio of 2.16 (95% CI, 1.21–3.88; log-rank *p* = 0.008). Shaded areas represent 95% confidence intervals.

**Table 1:** Baseline characteristics of patients undergoing ptosis repair with intraocular surgery (PRIOS) vs. ptosis repair alone (PRO), before and after propensity score matching.

		Patients, no (%)					
		Before propensity score matching			After propensity score matching^a^		
Characteristic	ICD-10/CPT/TriNetX code	PRIOS (*N* = 1602)	PRO (*N* = 12,093)	SMD	PRIOS (*N* = 1598)	PRO (*N* = 1598)	SMD
Age at Index, mean (SD), years	NA	71.8 ± 6.9	69.4 ± 8.4	0.320	71.8 ± 6.9	72.1 ± 7.3	0.042
Sex	NA						
Female	NA	1049 (65.5)	7675 (63.5)	0.042	1045 (65.4)	1031 (64.5)	0.018
Male	NA	537 (33.5)	3977 (32.9)	0.013	537 (33.6)	553 (34.6)	0.021
Race							
White	2106-3	1265 (79.0)	9388 (77.6)	0.032	1262 (79.0)	1310 (82.0)	0.076
Black or African American	2054-5	90 (5.6)	734 (6.1)	0.019	90 (5.6)	85 (5.3)	0.014
Asian	2028-9	64 (4.0)	469 (3.9)	0.006	63 (3.9)	59 (3.7)	0.013
Ethnicity							
Hispanic or Latino	2135-2	144 (9.0)	818 (6.8)	0.083	144 (9.0)	120 (7.5)	0.055
Essential (primary) hypertension	I10	946 (59.1)	5,926 (49.0)	0.203	942 (58.9)	953 (59.6)	0.014
Hyperlipidaemia, unspecified	E78.5	721 (45.0)	4534 (37.5)	0.153	719 (45.0)	741 (46.4)	0.028
Type 2 diabetes mellitus	E11	497 (31.0)	2706 (22.4)	0.196	493 (30.9)	486 (30.4)	0.010
Overweight and obesity	E66	329 (20.5)	2033 (16.8)	0.096	327 (20.5)	317 (19.8)	0.016
Anxiety, dissociative, stress-related, somatoform and other nonpsychotic mental disorders	F40-F48	327 (20.4)	2081 (17.2)	0.082	326 (20.4)	319 (20.0)	0.011
Systemic connective tissue disorders	M30-M36	85 (5.3)	405 (3.3)	0.096	84 (5.3)	85 (5.3)	0.003
Dry eye syndrome	H04.12	565 (35.3)	2615 (21.6)	0.306	561 (35.1)	553 (34.6)	0.011
Blepharitis	H01.0	156 (9.7)	864 (7.1)	0.093	154 (9.6)	157 (9.8)	0.006
Contact Lens Services	1012841	33 (2.1)	190 (1.6)	0.037	33 (2.1)	21 (1.3)	0.058
Nicotine dependence	F17	103 (6.4)	644 (5.3)	0.047	101 (6.3)	85 (5.3)	0.043
Problems related to housing and economic circumstances	Z59	10 (0.6)	32 (0.3)	0.054	10 (0.6)	10 (0.6)	0.000
Problems related to social environment	Z60	12 (0.7)	21 (0.2)	0.085	10 (0.6)	10 (0.6)	0.000

*CPT* current procedural terminology, *ICD-10* international classification of diseases, tenth revision, *SMD* standardized mean difference.

^a^Propensity score matching (PSM) was conducted on the following baseline characteristics: age at index, sex, race, ethnicity, essential (primary) hypertension, type 2 diabetes mellitus, hyperlipidaemia, overweight and obesity, systemic connective tissue disorders, blepharitis, dry eye syndrome, anxiety, dissociative, stress-related, somatoform and other nonpsychotic mental disorders, nicotine dependence, problems related to housing and economic circumstances, problems related to social environment, and contact lens services.

This multicentre retrospective cohort study found that intraocular surgery following ptosis repair is associated with a significantly increased risk of reoperation, with a sustained effect at 2, 5, and 10 years. The observed twofold increase aligns with prior evidence implicating intraocular surgery in ptosis repair failure. A recent multivariate analysis identified intraocular surgery as a potential risk factor for ptosis repair failure; however, its findings were limited by low power and a broad failure definition that included patient or physician dissatisfaction, potentially capturing cosmetic dissatisfaction rather than clinically significant recurrence requiring reoperation [2].

Kaplan–Meier analysis showed higher cumulative reoperation incidence in the PRIOS group, with earlier onset (∼3 months vs ∼6 months), supporting the hypothesis that intraocular surgery may compromise repair durability. Despite the elevated relative risk, the absolute reoperation rate remained low, underscoring clinical relevance while warranting measured interpretation during patient counselling.

Study limitations include the retrospective design, lack of laterality data, and absence of surgical detail or procedure stratification. Nonetheless, the study is strengthened by a large, matched cohort, extended follow-up, and the use of reoperation as a specific, objective outcome reflective of clinically significant recurrence.

In conclusion, intraocular surgery following ptosis repair is associated with a significantly increased risk of reoperation. These findings support incorporating recurrence risk into preoperative discussions and may inform surgical sequencing in patients undergoing both eyelid and intraocular procedures.

## Summary

### What was known before

- Acquired blepharoptosis can recur after surgical correction, influenced by surgical technique, patient factors, and ocular comorbidities.
- Intraocular surgery is a recognized cause of new-onset ptosis due to mechanical and anesthetic factors.
- Prior studies have suggested an association between intraocular surgery and ptosis repair failure, though evidence has been limited and methodologically heterogeneous.

### What this study adds

- Intraocular surgery after ptosis repair is associated with a significantly increased risk of reoperation for recurrent ptosis, with hazard ratios of 2.16 at 2 years, 1.98 at 5 years, and 1.67 at 10 years.
- Kaplan–Meier curves show earlier and sustained separation in the PRIOS group, suggesting reduced repair durability following intraocular surgery.
- The association persists despite low absolute reoperation rates, emphasizing clinical significance while supporting balanced patient counseling.
- This is the largest study to date to quantify the long-term risk of ptosis repair failure following intraocular surgery using a matched real-world cohort and objective surgical outcome.

## Supplementary information

Supplementary Material

## Data Availability

The data that support the findings of this study are available from TriNetX, LLC but third-party restrictions apply to the availability of these data. The data were used under license for this study with restrictions that do not allow for the data to be redistributed or made publicly available. However, for accredited researchers, the TriNetX data is available for licensing at TriNetX, LLC. Data access may require a data sharing agreement and may incur data access fees.
