# Supplementary material for: Risk of reoperation for recurrent ptosis following intraocular surgery
Source: Eye (Lond). 2025 Jun 16;39(11):2314–7. doi: 10.1038/s41433-025-03883-2 (PMC12274518; doi:10.1038/s41433-025-03883-2)
Supplement: Supplementary file 1 — Supplementary Material [file 41433_2025_3883_MOESM1_ESM.docx]

**Supplementary Material**

**Supplementary Methods**

This retrospective cohort study was conducted using the TriNetX global health research network, which provides access to deidentified electronic medical records from over 160 million patients across 141 healthcare organizations, primarily in the United States and Western Europe. Data available through the platform include diagnoses, procedures, medications, laboratory results, and demographics.

All analyses were performed in March 2025 using the Global Collaborative Network, restricted to patients with an index event within the past 20 years per TriNetX platform limitations. The TriNetX LIVE™ platform performs analyses within the secure environments of each participating institution and returns only aggregate-level results to the researcher. Statistical computations were executed using Java 11.0.16 (Apache Commons Math 3.6.1), R 4.0.2 (Hmisc 1-1, Survival 3.2-3), and Python 3.7 (lifelines 0.22.4, matplotlib 3.5.1, numpy 1.21.5, pandas 1.3.5, scipy 1.7.3, statsmodels 0.13.2). The study adhered to all relevant data protection standards, including HIPAA and the EU General Data Protection Regulation (GDPR), and was exempt from Institutional Review Board approval due to the use of deidentified data. TriNetX is ISO 27001:2013 certified.

Patients were identified using Current Procedural Terminology (CPT) and International Classification of Diseases, Tenth Revision (ICD-10) codes. The Ptosis Repair with Intraocular Surgery (PRIOS) cohort included patients aged ≥50 years who underwent ptosis repair (CPT 67901–67908), followed by their first recorded intraocular anterior or posterior segment surgery (CPT 1009892, 1009771) within 1 month to 10 years after the eyelid procedure. The Ptosis Repair Only (PRO) cohort included patients with the same inclusion criteria for ptosis repair but no subsequent intraocular surgery recorded during the 10-year follow-up.

To reduce potential confounding from unrelated conditions that may affect eyelid anatomy or function, we excluded patients with diagnoses of myoneural junction or muscle disease (ICD-10 G70–G73), third nerve palsy (H49.0), Horner’s syndrome (G90.2), periocular trauma (S01.1), ocular or orbital injury (S05), burns involving the head or face (T20), and congenital anomalies of the eyelid, lacrimal system, or orbit (Q10). All patients were required to have at least one year of follow-up after ptosis repair.

Baseline covariates were assessed within the 12 months prior to the index event (intraocular surgery for PRIOS; ptosis repair for PRO). Propensity score matching was performed using logistic regression with 1:1 greedy nearest neighbor matching and a caliper width of 0.1 pooled standard deviations of the logit of the propensity score. Covariates included age, sex, race, ethnicity, and comorbidities: essential hypertension, hyperlipidemia, type 2 diabetes mellitus, overweight and obesity, anxiety and stress-related disorders, systemic connective tissue disorders, dry eye syndrome, blepharitis, contact lens use, nicotine dependence, housing and economic problems, and social environment challenges.

The primary outcome was reoperation for recurrent ptosis, defined by repeat ptosis surgery (CPT 67901–67908) occurring at least 3 months after the index event to avoid capturing immediate postoperative revisions. Patients were followed for up to 2 years, with sensitivity analyses extending follow-up to 5 and 10 years. Kaplan–Meier survival curves, log-rank tests, and Cox proportional hazards models were used to estimate cumulative incidence and hazard ratios. Proportionality was assessed via Schoenfeld residuals.

**Supplementary Figure 1.** Kaplan–Meier Analysis of Reoperation for Recurrent Ptosis (5-Year Follow-up)


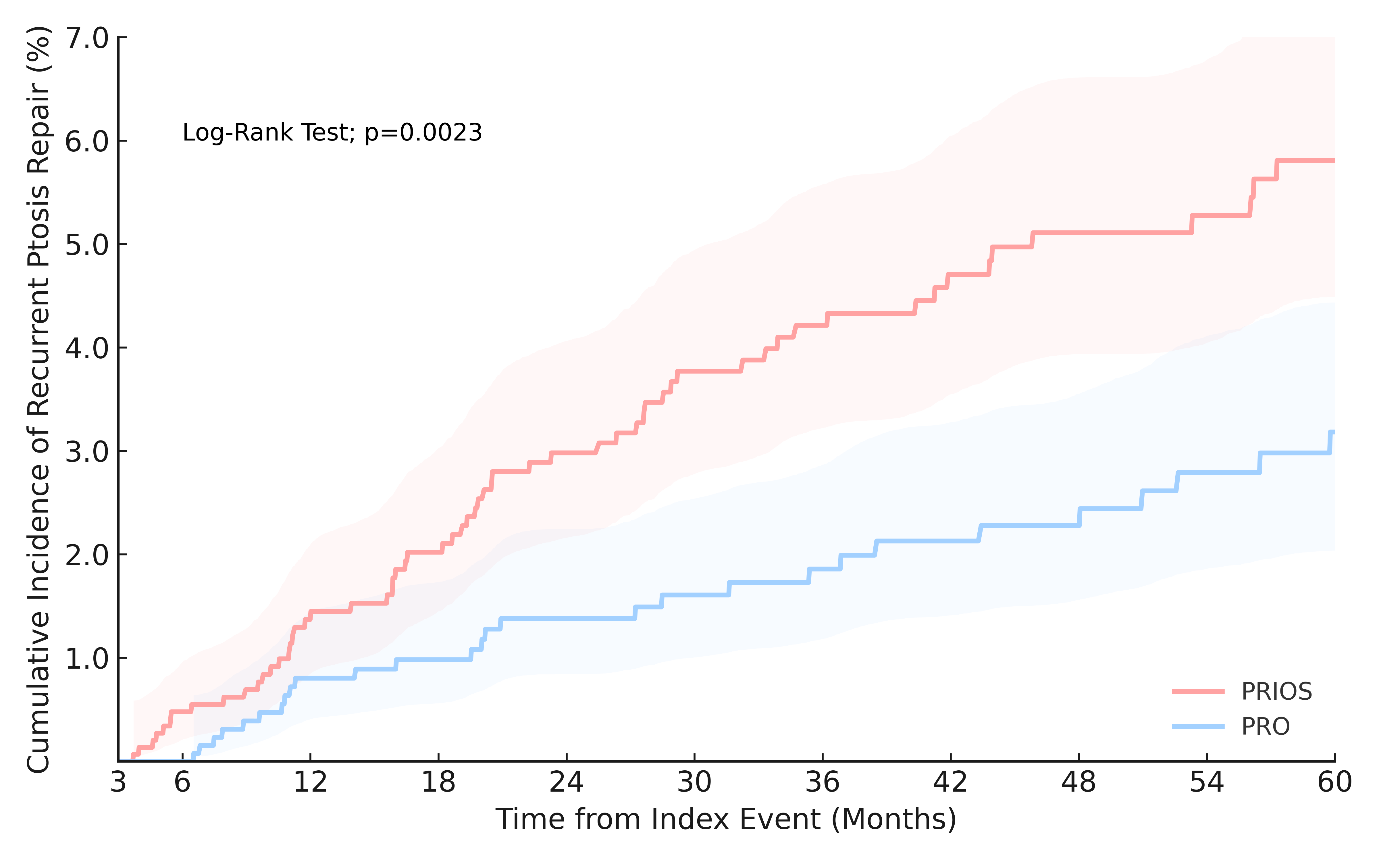


Legend: This Kaplan–Meier curve shows the cumulative incidence of reoperation for recurrent ptosis over 60 months in the PRIOS (Ptosis Repair with Subsequent Intraocular Surgery) and PRO (Ptosis Repair Only) cohorts. The PRIOS cohort (red) demonstrated a higher cumulative incidence than the PRO cohort (blue), with a hazard ratio of 1.98 (95% CI, 1.27–3.10; log-rank p = 0.0023). Shaded areas indicate 95% confidence intervals.


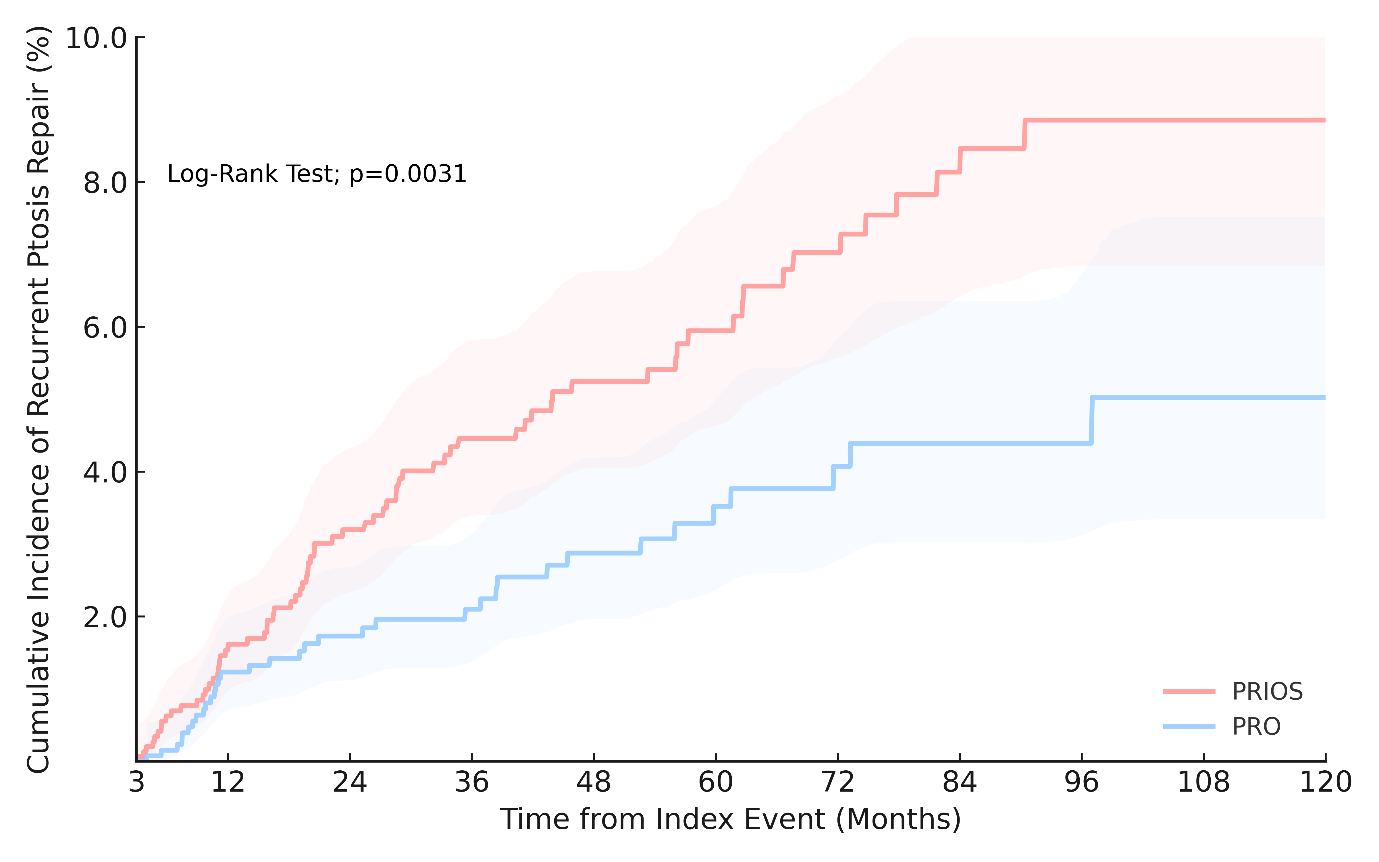
**Supplementary Figure 2.** Kaplan–Meier Analysis of Reoperation for Recurrent Ptosis (10-Year Follow-up)

Legend: This Kaplan–Meier curve shows the cumulative incidence of reoperation for recurrent ptosis over 120 months in the PRIOS (Ptosis Repair with Subsequent Intraocular Surgery) and PRO (Ptosis Repair Only) cohorts. The PRIOS cohort (red) demonstrated a higher cumulative incidence than the PRO cohort (blue), with a hazard ratio of 1.67 (95% CI, 1.12–2.48; log-rank p = 0.0031). Shaded areas indicate 95% confidence intervals.
